# Editorial: Soil biodiversity and regenerative agriculture: the path to achieve SDGs

**DOI:** 10.3389/fmicb.2025.1755391

**Published:** 2026-01-22

**Authors:** Anukool Vaishnav, Durgesh Kumar Jaiswal, Jagajjit Sahu

**Affiliations:** 1 Laboratory of Fungal Biology, Institute of Microbiology, Czech Academy of Sciences, Prague, Czechia; 2 Department of Biotechnology, GLA University Mathura, Mathura, India; 3 Department of Biosciences, Graphic Era (Deemed to be University), Dehradun, India; 4 Bioinformatics Nexus Network (BNsquare), GyanArras Academy, Bhubaneswar, India

**Keywords:** abiotic stress, biofertilizers, biopesticides, biotic stress, regenerative agriculture, soil biodiversity, sustainable developmental goals (SDGs)

## Background

**Figure 1:**
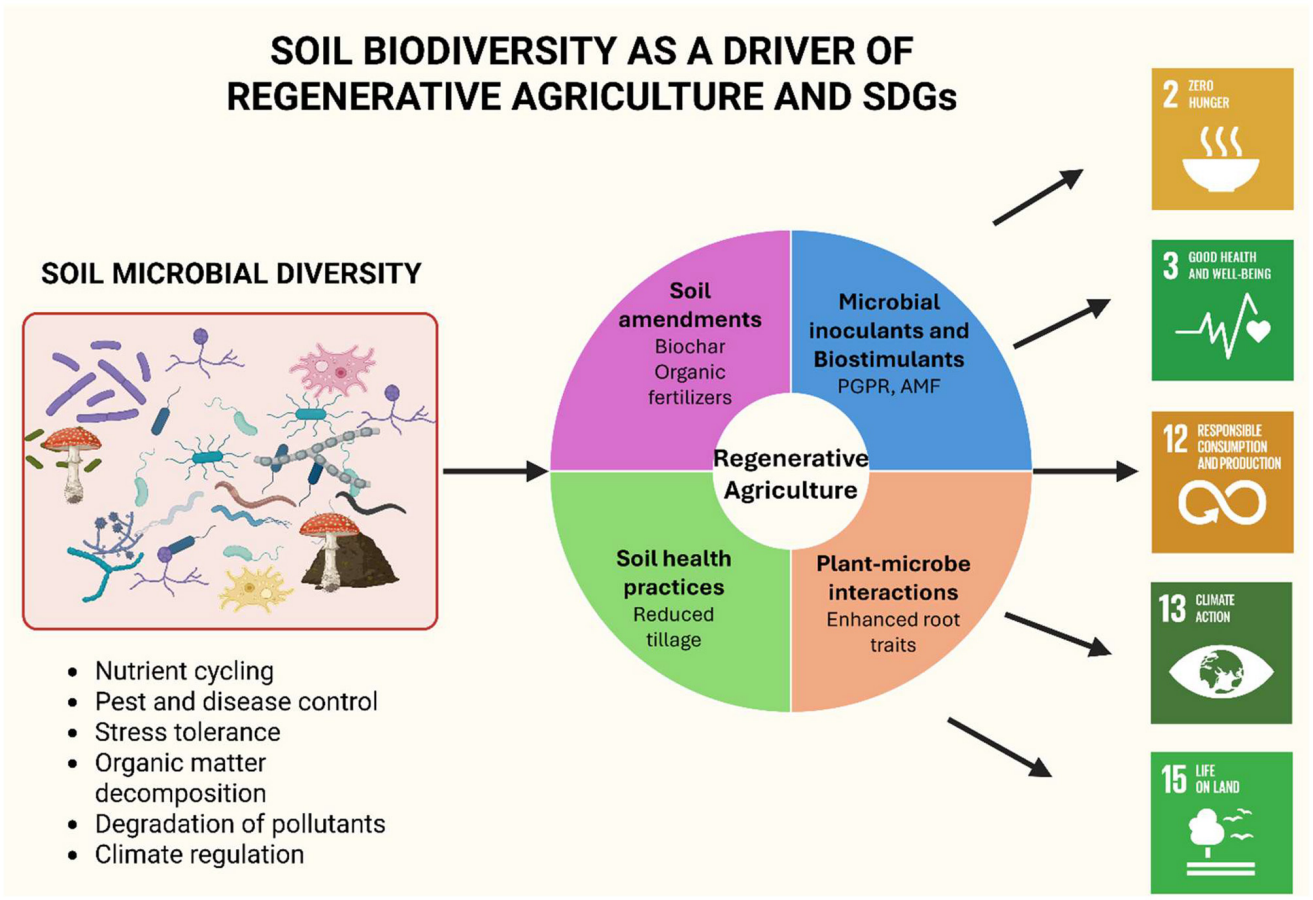
Conceptual framework illustrating how soil microbial diversity underpins regenerative agriculture and supports progress toward key Sustainable Development Goals (SDGs). The diagram presents a three-part pathway. On the left- major components of soil biodiversity including bacteria, fungi, actinomycetes, nematodes, protozoa and soil organic matter, are shown as the foundation of essential ecosystem processes such as nutrient cycling, pest and disease suppression, organic matter mineralization and stress mitigation. Arrows led to the central panel- summarizes core elements of regenerative agriculture: microbial inoculants and biostimulants, soil amendments such as biochar and organic fertilizers, practices that enhance soil health and carbon dynamics and plant-microbe interactions that improve nutrient uptake and stress tolerance. Together, these processes strengthen crop resilience and increase soil function. The right panel- links these improvements to five SDGs most directly influenced by regenerative agricultural management: SDG 2 (Zero Hunger), SDG 3 (Good Health and Wellbeing), SDG 12 (Responsible Consumption and Production), SDG 13 (Climate Action) and SDG 15 (Life on Land).

The soil is an ever-changing biological ecosystem, harbor microorganisms that help in nutrient cycling, and are necessary for plant development, disease resistance, and carbon storage. The intensive farming practices of the past decades have completely disrupted these functions by applying large amounts of fertilizers and pesticides, and by carrying out the kind of practices that lower the amount of organic matter and biodiversity in the areas concerned (Pedrinho et al., 2024). Hence, restoring soil biological integrity is a must for regenerative agriculture (Schreefel et al., 2020). Figure 1 illustrating soil biodiversity as crucial for regenerative agriculture and SDGs.

The compilation of this Research Topic inmicrobes ande different ways in which microbes help to make nutrients available and to tolerate stress, look into the effects of different biofertilizers and microbial consortia developed for particular crops, and use omics technologies for revealing the interactions between plants and microbes, and contemplate how these findings might be applied on a large scale in regenerative practices. All the studies together suggest crucial approaches that can use to meet up different SDGs: zero hunger (SDG 2), health and wellbeing (SDG 3), responsible consumption and production (SDG 12), climate change mitigation (SDG 13), and preserving terrestrial ecosystems (SDG 15).

## Contributions

This Research Topic comprises six original research articles that each address different facets of soil biodiversity in agricultural contexts. Below, we discuss key contributions and their broader implications.

1. Mitigating salinity in mustard via *Bacillus flexus*

The research by Singh and Prasad elaborates the usage of *Bacillus flexus*, a salt-tolerant PGPR, in improving mustard (*Brassica juncea*) growth under salt stress. The inoculation with *B. flexus* resulted in enhance plant development in terms of germination, biomass, leaf area, chlorophyll content, and enzyme activities. Moreover, the bacterial inoculation also showed a reduction in oxidative stress, electrolyte leakage and malondialdehyde levels. Thus, these results are pointed at the deployment of stress-tolerant microbial formulations to restore saline soils and increase plant productivity.

2. Soil amendments to enhance alfalfa performance under salinity

The study by Jabborova et al. focused on how different soil amendments such as biochar, hydrogel, and biofertilizer affect alfalfa in the salinity-prone areas of the Aral Sea. Three-fold of plant biomass was increased with biochar and biofertilizer application, with biochar being particularly effective in chlorophyll content. Furthermore, the amendments were beneficial in terms of soil quality also, enriched soil nitrogen and phosphorus content. This finding demonstrates the effectiveness of the organic carbon and microbial input strategy that could lead to abiotic stress resilience, a defining characteristic of regenerative agriculture.

3. Biocontrol of tomato wilt using PGPB

Study by Ansari et al. demonstrated the suppressive effect of four bacteria on Fusarium wilt in tomato plants: *Pseudomonas aeruginosa, P. putida, Paenibacillus polymyxa*, and *Bacillus cereus*. The bacterial-inoculated plants showed enhanced antioxidant enzyme activities, the up-regulation of defense-related genes, and reduced disease severity. The highest effect was seen with P. polymyxa inoculated plants. This research shows how microbial biocontrol agents can be one of the ways to reduce the use of chemical fungicides and act as the agents of change for environmentally friendly cropping systems; hence, they contribute to SDG 3 and SDG 15.

4. Carbon source management to improve rose yield and quality

Li et al. conducted a year-long field trial with a red-soil ecosystem to investigate the effects of biochar and organic fertilizer on the soil microbial communities and cut flower production of rose. The application of biochar induced population of bacterial taxa such as *Sphingomonas* which are beneficial for plant growth, whereas organic fertilizer elevated microbial activity and nutrient availability. Both treatments were effective in increasing flower yield and quality. The findings of this research highlight the significance of controlling carbon inputs in determining the soil microbial community and favorable crop outcomes, thus soil biodiversity being the key player in the regenerative model.

5. Microbial correlations of wine grape quality

Guo et al. applied detailed sequencing methods to figure out the connections between microbiota in the roots and rhizosphere and fruit chemistry of three wine grape cultivars. They found that certain microbial groups were closely linked with essential quality traits, for instance, associations between Mortierellomycota and malic acid, *Aphelidiomyceta* and flavonols, and several bacterial genera with tannin content. Using soil and root microbiomes as indicators for plant health and biochemical traits that affect fruit and beverage quality, this study links soil biodiversity with SDG 2 as well as SDG 12.

6. Endophytic fungi in *Paris polyphylla* seeds during storage

Peng et al. analyzed changes of endophytic fungal communities during seed storage in *Paris polyphylla* var. *chinensis* and studied their reaction to phytohormones in the initial developmental stage of the seedling. The research showed that storage changed the fungal diversity and composition, and some fungi reacted intensively to hormone treatments. This finding consents to the conservation of seed and soil-associated microbial diversity as the source of propagation.

## Conclusion and future directions

This Research Topic exemplify the variability of soil microbial communities in their different roles in regenerative agriculture. Firstly, microbial behavior depends on the location, the carbon content into the soil, the type of crop planted, and the stress conditions in the vicinity. The proper management of these factors can promote beneficial plant-microbe interaction for both agricultural productivity and ecosystem functioning. Secondly, microbe-based approaches benefit in chasing SDGs of multiple areas like lower disease incidence, enhance yield even under unfavorable conditions, and increase the quality of food. Thirdly, the development of high-throughput sequencing can relate microbial community profiles to functional outcomes leading to more precise and effective interventions.

Nevertheless, these developments have not conquered significant difficulties yet. The beneficial microbes have to be consistent across diverse and often stress environments, and also their application will have to be controlled by appropriate regulations and the setting up of cost-effective delivery mechanisms. Long-term field trials are required to confirm the effects on yield, soil health, and ecosystem services over time. Measures like payments for ecosystem services, soil health incentives, and certification schemes will be policy mechanisms that help greatly in this regard.

## References

[B1] Pedrinho A. Mendes L. W. de Araujo Pereira A. P. Araujo A. S. F. Vaishnav A. Karpouzas D. G. . (2024). Soil microbial diversity plays an important role in resisting and restoring degraded ecosystems. Plant Soil 500, 325–349. doi: 10.1007/s11104-024-06489-x

[B2] Schreefel L. Schulte R. P. De Boer I. J. M. Schrijver A. P. Van Zanten H. H. E. (2020). Regenerative agriculture–the soil is the base. Glob. Food Sec. 26:100404. doi: 10.1016/j.gfs.2020.100404

